# Significance of Polymorphisms and Expression of Enzyme-Encoding Genes Related to Glutathione in Hematopoietic Cancers and Solid Tumors

**DOI:** 10.1155/2015/853573

**Published:** 2015-11-23

**Authors:** Szymon Zmorzyński, Grażyna Świderska-Kołacz, Dorota Koczkodaj, Agata Anna Filip

**Affiliations:** ^1^Department of Cancer Genetics and Cytogenetics Laboratory, Medical University of Lublin, 20-080 Lublin, Poland; ^2^Department of Animals Physiology, The Jan Kochanowski University in Kielce, 25-406 Kielce, Poland

## Abstract

Antioxidant compounds such as glutathione and its enzymes have become the focus of attention of medical sciences. Glutathione, a specific tripeptide, is involved in many intercellular processes. The glutathione concentration is determined by the number of GAG repeats in gamma-glutamylcysteine synthetase. GAG polymorphisms are associated with an increased risk of schizophrenia, berylliosis, diabetes, lung cancer, and nasopharyngeal tumors. Cancer cells with high glutathione concentration are resistant to chemotherapy treatment. The oxidized form of glutathione is formed by glutathione peroxidases (GPXs). The changes in activity of GPX1, GPX2, and GPX3 isoforms may be associated with the development of cancers, for example, prostate cancer or even colon cancer. Detoxification of glutathione conjugates is possible due to activity of glutathione S-transferases (GSTs). Polymorphisms in GSTM1, GSTP1, and GSTO1 enzymes increase the risk of developing breast cancer and hepatocellular carcinoma. Gamma-glutamyl transpeptidases (GGTs) are responsible for glutathione degradation. Increased activity of GGT correlates with adverse prognosis in patients with breast cancer. Studies on genes encoding glutathione enzymes are continued in order to determine the correlation between DNA polymorphisms in cancer patients.

## 1. Introduction

Carcinogenesis is a multistage, complex process, mostly related to DNA mutations. The factors taken into account are mainly external factors, namely, ionizing radiation (*X*, *γ*) and nonionizing radiation (UV), as well as internal (individual) factors. The latter include impairment of cell division control systems, immunological control systems, and incorrect function of antioxidation mechanisms. Oxidation of nitrogenous bases in DNA accelerates and increases the probability of malignant transformation. Oxidation of protein thiol groups (-SH) alters the properties of transcription factors and histones. On the one hand, oxidative stress can induce carcinogenesis, but on the other hand it may have an anticancer effect, for instance by inducing apoptosis [1]. The impact of oxidative stress on cells can be acute or chronic. Chronic oxidative stress may cause slight DNA damage that is accumulated. Changes in DNA caused by action of reactive oxygen species can lead to the following tumors: solid tumors (e.g., prostate cancer, and melanoma) and hematologic malignancies (e.g., acute lymphocytic leukemia, myelodysplastic syndrome, chronic myelogenous leukemia, and acute myelogenous leukemia) [2].

Glutathione (GSH), referred to as gamma-glutamyl-cysteinylglycine, is a tripeptide formed from glutamic acid, cysteine, and glycine. Glutamic acid forms a specific gamma-peptide bond with cysteine. The antioxidant function of glutathione is conditioned on the presence of cysteine thiol groups that have oxidation-reduction properties [3]. Inside the cells, cysteine mainly occurs in the sulfhydryl form, while in extracellular fluid it forms disulfides [4]. Glutathione is not only the first defense line against oxidative stress, but it is also involved in a number of metabolic processes such as regulation of enzyme activity, gene expression, signal transduction, and intensification of cytoplasmic and transmembrane transport. The main role of glutathione is detoxification of xenobiotics and some endogenous compounds [5].

Glutathione is present in cells and body fluids such as blood [6]. Inside the cells, glutathione is present in cytosol and organelles, endoplasmic reticulum, nucleus, and mitochondria [7, 8]. Under physiological conditions, over 98% of intracellular glutathione is in the reduced thiol form (GSH). The remainder is mostly disulfide form (GSSG) and mixed disulfides (mainly GS-S-protein) [6]. Under normal conditions, GSSG is only found in higher concentrations than GSH in the endoplasmic reticulum [7, 8].

Detoxification of xenobiotics, that is, GSH conjugates, with endogenous or exogenous electrophilic compounds is possible due to the activity of glutathione S-transferases (GSTs), which belong to the family of Phase II detoxification enzymes [9]. Oxidation of protein sulfhydryl groups affects enzyme activity, intracellular and transmembrane transport, signal transduction, cell cycle progression, and gene expression. The latter process is regulated by transcription factors that are sensitive to changes in cellular redox status, for example, TP53. This protein has 12 cysteine residues, and oxidation of some of them inhibits its activity [10].

The oxidized form of glutathione is formed either by glutathione peroxidases (GPXs) or as a result of direct reaction of GSH with electrophilic compounds [6, 11]. Glutathione peroxidases are a family of enzymes that reduce hydrogen peroxides and other organic peroxides [12]. In cells, these enzymes are active in cytosol and mitochondria or may be bound with the inner surface of the cell membrane [12].

Reduced glutathione is degraded to glutamic acid and cysteinyl-glycine in the process catalyzed by gamma-glutamyl transpeptidase (GGT). This enzyme is located on the outer surface of the cell membrane. Cysteinyl-glycine is hydrolysed by dipeptidases and resulting amino acids are used again for the synthesis of glutathione. GGT can also decompose a form of GSSG and GSH conjugates [13]. The latter are excreted into bile or transformed into mercapturic acid and removed to urine [14]. Increased activity of GGT in serum is associated with liver diseases, excessive alcohol consumption, stroke, diabetes, and lung, liver, prostate, or breast cancers [15–17].

## 2. Glutathione and Synthesizing Enzymes

In recent years, the key role of oxidative stress in pathogenesis has been increasingly emphasized, for example, in cardiovascular disease [18, 19], fatty liver disease [20], spinal cord injury [21], Alzheimer's disease [22], and schizophrenia and autism [23]. Antioxidant compounds, including glutathione, have become the focus of attention of medical sciences.

Glutathione is synthesized in two steps in ATP-dependent processes. The first step is catalyzed by gamma-glutamylcysteine synthetase (GCL), which consists of two subunits, the first is catalytic GCLC (encoded by the* GCLC* gene,* locus* 6p12) and second is modifying GCLM (encoded by the* GCLM* gene,* locus* 1p22.1). GCL synthesizes an atypical peptide bond between the carboxyl group of gamma-glutamic acid and the amino group of cysteine [24]. The second step in the synthesis of glutathione is catalyzed by glutathione synthetase (GS) encoded by the* GSS* gene (*locus* 20q11.2) [3, 24].

Reducing the amount of ATP inhibits the synthesis of GSH. This process depends inter alia on the availability of starting materials, amounts of the two different subunits of the enzyme GCL, and a negative feedback mechanism by GSH to activity of GCL [25–27].

High concentration levels of GSH observed in many tumors are associated with increased GCL activity [28]. The GSH concentration and GCL activity are affected by the number of GAG trinucleotide repeats in the 5′ untranslated region (5′-UTR) of the* GCLC* gene [29]. Polymorphism of nucleotide triplets is associated with the occurrence of five alleles containing 4, 7, 8, 9, and 10 GAG repeats,* GAG-4, GAG-7, GAG-8, GAG-9*, and* GAG-10*, respectively. The function of GAG triplets of the 5′-UTR involves inhibition of translation [30]. People with the* GAG-9/9* genotype (two alleles have nine GAG repeats in the 5′-UTR) have lower concentration of GSH than those with the* GAG-7/9* and* GAG-7/7* genotypes. Polymorphisms of* GAG-4* and* GAG-10* are rare [30]. GAG polymorphisms are associated with an increased risk of certain diseases, including schizophrenia, berylliosis, diabetes, lung cancer, and nasopharyngeal tumors [30]. Nichenametla et al. demonstrated that the* GAG-7/7* genotype is associated with increased incidence of lung and nasopharyngeal tumors [30]. It has been found that patients with lung cancer or nasopharyngeal cancer with* GAG-7/7* genotype have survival times that are one year shorter than in other genotypes [30].

Tumor cells with a high concentration of GSH are resistant to chemotherapy treatment [28]. High concentration levels of GSH have been observed in patients with liver cancer and melanoma. Positive correlation between concentration of GSH and proliferation of cancer cells and metastasis was also demonstrated [28]. Increased levels of GSH are also observed in normal cells such as neurons, lung cells, heart cells, and blood vessel cells [31].

## 3. Glutathione Peroxidases

The family of glutathione peroxidases is derived from a single enzyme containing cysteine in the active-center [32]. In human cells, there are eight genes identified that encode distinct isoforms of GPX:* GPX1* (*locus* 3p21.3),* GPX2* (*locus* 14q24.1),* GPX3* (*locus* 5q23),* GPX4* (*locus* 19p13.3),* GPX5* (*locus* 6p22.1),* GPX6* (*locus* 6p22.1),* GPX7* (*locus* 1p32), and* GPX8* (*locus* 5q11.2) [33]. So far, it has been shown that changes in the activity of the GPX1, GPX2, and GPX3 isoforms may be associated with the development of cancer [12].

The activity of GPX1 can prevent DNA damage and inhibit synthesis of inflammatory mediators such as prostaglandins and leukotrienes [12]. There is a relationship between the activity of GPX1 and concentrations of selenium binding protein (SBP1). SBP1 reduces the activity of GPX1. In turn, GPX1 inhibits SBP1 expression at the transcriptional level, as well as by epigenetic modifications [33]. In patients with hepatocellular carcinoma, low SBP1 levels are associated with high activity of GPX1, metastasis, and shorter survival time [34]. Similar observations were seen in patients with prostate cancer [35].

Overexpression of the* GPX2* gene is observed in many diseases, mainly in cancers, such as colon cancer, squamous cell carcinoma, and pulmonary adenocarcinoma, as well as in premalignant lesions like Barrett's esophagus [12].

Reduced GPX3 expression has been found in prostate cancer, in endometrial cancer, and in head and neck cancer as well. Hypermethylation of the* GPX3* promoter reduces expression of this gene, which is associated with the mechanism of drug resistance and the shorter survival of cancer patients [12, 36]. Interaction between GPX3 and the PIG3 protein (a protein induced by TP53 of gene 3) activates the apoptosis in prostate cancer cells [37]. Many chemotherapeutic agents generate free radicals and thus induce apoptosis in tumor cells [12, 36].

## 4. Glutathione S-Transferases

These enzymes are present in cytosol and mitochondria; they may be associated with cell membrane and they influence several intracellular processes related to stress response, cell proliferation, apoptosis, neoplastic transformation, metastatic tumor formation, and development of drug resistance mechanisms [38]. The superfamily of genes encoding cytosolic glutathione S-transferases consists of eight classes: *α* (*GSTA*), *κ* (*GSTK*), *μ* (*GSTM*), *ω* (*GSTO*), *π* (*GSTP*), *ς* (*GSTS*), *θ* (*GSTT*), and *ζ* (*GSTZ*) [9]. This classification was established based on immunological properties and catalytic activity of enzymes, amino acid sequence homology, and the sequence of bases in genes, as well as the location of genes on chromosomes. There are single nucleotide polymorphisms (SNP) occurring within genes that encode glutathione S-transferases, which are associated with an increased risk of cancer and diverse response to treatment. For example, polymorphisms in the* GSTM1* and* GSTP1* genes increase the risk of developing breast cancer and hepatocellular carcinoma [39, 40]. Under stress conditions, GST isoenzymes cause the release of kinases and induce apoptosis [41].

Glutathione S-transferases are encoded by 16 genes. Most data in the literature refer to polymorphisms of the following transferases: GSTM1 (encoded by the* GSTM1* gene,* locus* 1p13.3), GSTP1 (encoded by the* GSTP1* gene,* locus* 11q13), and GSTT1 (encoded by the* GSTT1* gene,* locus* 22q11.23) [9, 41–43]. In the* GSTM1* and* GSTT1* genes, there are null genotypes (deletions of both alleles) often observed, which occur in Caucasian populations with an incidence of 42–60% and 13–26%, respectively [43]. No evidence of enzyme activity encoded by the* GSTM1* and/or* GSTT1* genes is found in approximately 20–50% of people, due to deletion of both alleles. DNA of null genotype cells is more likely to be subject to damage caused by carcinogens [9]. The meta-analysis carried out by He et al. showed that the deletion of both alleles of* GSTT1* increases the risk of chronic lymphocytic leukemia [9]. Furthermore, double null genotype of* GSTM1* and* GSTT1* may be associated with the development of chronic lymphocytic leukemia [9, 42, 43].

The best known polymorphism in the pi-class of genes encoding glutathione S-transferases (GSTP) is described in the* GSTP1* gene, wherein substitution from A to G at codon 105 (A105G) occurs. This substitution reduces the ability of GSTP1 to bind to certain mutagens, which increases the amount of DNA damage and cancer risk [44]. The A105G substitution in the* GSTP1* gene reduces the activity of the GSTP1 enzyme [9]. Voso et al. have demonstrated a correlation between the substitution in the* GSTP1* gene (A105G) and a longer progression-free period in patients with acute myelogenous leukemia [41].

The activity of glutathione S-transferases alpha (GSTA) was found in liver, kidney, lung, stomach, testes, and ovaries [45]. The GSTA class of enzymes is encoded by five genes (*GSTA1–5*) located at chromosome 6 (*locus* 6p12). There are seven pseudogenes within this class [9]. Posttranslational modifications of GSTA1 and GSTA3 contribute to change in the properties of these enzymes and cause them to have isomerase activity, which is essential for the synthesis of steroid hormones in ovaries, testicles, and adrenal glands [46]. At least five polymorphisms in the promoter region of the* GSTA1* gene were described. This gene has two alleles,* GSTA1* ^*∗*^ *A* and* GSTA1* ^*∗*^ *B* [47]. Gravina et al. demonstrated that the combination of* GSTA1* ^*∗*^ *B* and* GSTT1* homozygotes, with the simultaneous deletion of both alleles of* GSTM1*, is a risk factor for the development of schizophrenia [48]. Expression of the* GSTA* gene and activity of glutathione S-transferases alpha in the liver are regulated by many exogenous factors such as dexamethasone, phenobarbital, and other antioxidant compounds [45]. Hormones can modulate the activity of glutathione S-transferases; for instance, expression of GSTA-encoding genes is inhibited by thyroxin and triiodothyronine [45].

Glutathione S-transferases omega (GSTO) are encoded by two genes:* GSTO1* and* GSTO2*. Three polymorphisms within the GSTO class of enzymes were described, GSTO1^*∗*^A140D, GSTO1^*∗*^E155del, and GSTO2^*∗*^N142D [49]. Research results indicate that there is a relationship between the GSTO1^*∗*^A140D polymorphism and an increased risk of breast cancer, hepatocellular carcinoma, cholangiocarcinoma, urothelial cancer, acute lymphoblastic leukemia, and non-small-cell lung cancer [50]. No known relationship was confirmed between polymorphisms of the* GSTO1* and* GSTO2* genes and an increased risk of development of breast, thyroid, and colon cancer [51]. The results of research carried out by Safarinejad et al. suggest that polymorphism of the* GSTP1* gene and its combination with the* GSTM1* and* GSTT1* polymorphisms may be associated with an increased risk of bladder cancer [52]. Yang et al. demonstrated that a deletion of both alleles of* GSTT1* is a predisposition to the development of lung cancer in Asian populations [53]. In contrast to other GST enzymes, glutathione S-transferases omega have cysteine in the active site and may form disulfide bonds with GSH, which activates glutathione-dependent dehydroascorbate reductase and sulphotransferase. Moreover, the GSTO enzymes are involved in signal transduction [49]. Expression of the* GSTO* genes is observed in many normal tissues and organs, such as liver, colon, ovaries, pancreas, prostate, and spleen [49]. Overexpression of* GSTO* genes is related to the induction of apoptosis in tumor cells [49].

Most often, the polymorphisms that decrease the activity of glutathione S-transferases are associated with an increased risk of cancer development [28]. Overexpression of certain* GST* genes may affect the phenomenon of drug resistance. Glutathione S-transferases can combine with mitogen activated protein kinases (MAP/MAPK). Apoptosis-inducing MAP kinases are thus blocked and cannot be activated by compounds having anticancer properties [54].

## 5. Gamma-Glutamyl Transpeptidases

In contrast to GSH synthesis, the degradation thereof occurs extracellularly on the surface of cells that show expression of gamma-glutamyl transpeptidase (GGTs) [55]. Isoforms of that enzyme are encoded by the following genes:* GGT1* (*locus* 22q11.23),* GGT2* (*locus* 22q11.21),* GGT5* (*locus* 22q11.23),* GGT6* (*locus* 17p13.2), and* GGT7* (*locus* 22q11.22). The* GGT3* and* GGT4* alleles are pseudogenes. Gamma-glutamyl transferase regulates cysteine homeostasis and catalyzes the metabolism of endogenous GSH conjugates with xenobiotics [55]. Increased GGT activity occurs in ovary, colon, liver, and skin cancers, as well as in leukemia [28]. There is also a correlation observed between increased activity of GGT and the adverse prognosis in breast cancer patients. Processes of GSH synthesis and degradation are in balance and are subject to strict control [6].

The promoter region of genes encoding gamma-glutamyl transpeptidases and glutathione S-transferases has a site that binds transcription factors such as NF-*κ*B, AP-1, AP-2, and Nrf2/Keap1. Reactive oxygen species cause Keap1 to release the Nrf2 protein which changes the location from cytoplasmic to nuclear and combines with sequences of ARE. This increases the expression of the genes that encode ferritin, glutathione reductase, glutathione S-transferases, two subunits of gamma-glutamylcysteine synthetase, NAD(P)H-ubiquinone oxidoreductase, multidrug resistance protein, and heme oxygenase 1 [28]. In many tumors, increased levels of the Nrf2 protein are observed, which causes the development of the drug resistance mechanism [56].

## 6. Conclusion

The effect of oxidative stress on carcinogenesis has not been fully explained so far. Alterations of antioxidant mechanisms are factors that contribute to limiting carcinogenesis. Many primary tumors are characterized by high activity of antioxidant enzymes, which leads to tumor cell resistance to chemotherapy. Prooxidative therapies, that is, those that generate the development of oxidative stress, have been more and more frequently used in cancer treatment. A recent study in patients with lung cancer showed that administration of N-acetyl-L-cysteine and vitamin E contributes to tumor progression via stimulation of cell proliferation by reducing the quantity of oxygen free radicals, DNA damage, and expression of the* TP53* gene [57]. It is not known what role in these processes is played by molecular signaling pathways associated with glutathione and glutathione enzymes, which may provide new therapeutic targets in cancer patients in the future. Currently, GSH concentration-reducing chemical compounds are used in initial clinical tests in order to enhance the effectiveness of chemotherapeutic agents. An example of an inhibitor of GSH synthesis is buthione sulfoximine that affects the activity of GCL, a key enzyme involved in the synthesis of glutathione.

Studies on genes encoding glutathione enzymes are continued in order to determine the correlation between DNA polymorphisms and the prognosis in patients with cancer. New therapeutic strategies are still being sought, mainly in the cancer diseases with insufficient effectiveness of treatment, such as in acute myeloid leukemia. Recently developed molecular methods used in the study of polymorphisms or mutations in genes encoding enzymes related to glutathione can contribute to the identification of new prognostic or predictive factors in cancer patients. This implies a potential role of these methods in designing personalized therapies. Early prediction of the course of disease allows physicians to make appropriate treatment decisions.
